# Identification of a New Cell Population Constitutively Circulating in Healthy Conditions and Endowed with a Homing Ability Toward Injured Sites

**DOI:** 10.1038/srep16574

**Published:** 2015-11-12

**Authors:** Claudia Lo Sicco, Roberta Tasso, Daniele Reverberi, Michele Cilli, Ulrich Pfeffer, Ranieri Cancedda

**Affiliations:** 1 Department of Experimental Medicine (DIMES), University of Genova, Genova, 16132, Italy; 2 A.O.U. San Martino-IST, National Cancer Research Institute, Genova, 16132, Italy

## Abstract

Stem and progenitor cells are the critical units for tissue maintenance, regeneration, and repair. The activation of regenerative events in response to tissue injury has been correlated with mobilization of tissue-resident progenitor cells, which is functional to the wound healing process. However, until now there has been no evidence for the presence of cells with a healing capacity circulating in healthy conditions. We identified a rare cell population present in the peripheral blood of healthy mice that actively participates in tissue repair. These Circulating cells, with a Homing ability and involved in the Healing process (CH cells), were identified by an innovative flowcytometry strategy as small cells not expressing CD45 and lineage markers. Their transcriptome profile revealed that CH cells are unique and present a high expression of key pluripotency- and epiblast-associated genes. More importantly, CH-labeled cells derived from healthy Red Fluorescent Protein (RFP)-transgenic mice and systemically injected into syngeneic fractured wild-type mice migrated and engrafted in wounded tissues, ultimately differentiating into tissue-specific cells. Accordingly, the number of CH cells in the peripheral blood rapidly decreased following femoral fracture. These findings uncover the existence of constitutively circulating cells that may represent novel, accessible, and versatile effectors of therapeutic tissue regeneration.

All body tissues, particularly those characterized by a high cell turnover, depend on innate regenerative events in order to function properly^1^. Stem cells are clonogenic and self-renewable populations that can differentiate into multiple cell lineages^2^,^3^. The concept of adult stem cells intended as cell populations restricted to their own tissue has been challenged by reports indicating that these cells can be mobilized in response to a tissue damage^4^,^5^,^6^,^7^. A growing number of scientific reports indicates that under the influence of various pathological stimuli, tissue-specific and/or bone marrow-derived stem/progenitor cells are rapidly mobilized to the blood stream, playing a crucial role in the repair of solid organs, acting directly or enhancing the re-activation of resident stem cells^5^,^7^,^8^. Indeed, peripheral blood (PB) is an ideal alternative source for progenitor cells owing to the ease of cell retrieval and blood bank storage. Upon appropriate mobilization strategies, many stem/progenitor cells are hosted by PB, such as Hematopoietic Stem Cells (HSCs), the archetype resident stem cells used for transplantation therapies^9^, and Endothelial Progenitor Cells (EPCs), effectively involved in endothelial regenerative processes^10^. In this context, the existence, in the PB, of progenitors sharing the phenotypic characteristics of Mesenchymal Stem Cells (MSCs) is still questioned, due to the lack of standard set of criteria for their definition^11^. It’s likely that individual PB-derived progenitors detected by different experimental strategies are overlapping but indicated with different names. This contribute to increase the confusion regarding their exact identification^12^.

All stem/progenitor cells isolated so far from the peripheral blood have been isolated only in pathological conditions or following a mobilization procedure. Until now there has been no evidence for the existence of progenitor cell populations circulating in physiological conditions. Moreover, all stem/progenitor cells isolated from the PB presented restricted differentiation capacity^13^,^14^. Recently the presence, in the bone marrow and other adult organs, of very small-sized stem cells with pluripotent characteristics has been reported^15^,^16^. However, there is a lack of consensus on the real existence of such cell population^17^, which possibly derives from poorly reproducible flowcytometric analysis and isolation procedures^18^.

Using a functional flowcytometry strategy, we developed a reproducible system for the isolation of Circulating cells derived from the peripheral blood of healthy mice endowed with a Homing capacity and involved in the Healing process (CH cells). CH cells are small cells characterized by the lack of expression of the pan-hematopoietic CD45 antigen, of the markers expressed by differentiated blood cells, as well as of markers typically associated to well-defined progenitors circulating upon injury. The analysis of the global transcriptional profile of the purified CH cells revealed their uniqueness when compared to other cells characterized by varying stemness degree.

Furthermore, CH cells were demonstrated to be progenitors functionally involved in the endogenous reparative events. Taking advantage of an injury model able to repair itself under optimal conditions, such as the stabilized fracture healing model^19^, in which the injury signals are sufficient to enhance and direct endogenous reparative events, we demonstrated that systemically transplanted CH cells possessed the capacity to migrate toward wounded sites. More importantly, injected cells were able to integrate in wounded tissues and to appropriately differentiate into a broad spectrum of tissue-specific cells.

Collectively, our results support the idea that CH cells are key effectors of innate regenerative events and could open up a new way of approaching tissue regeneration.

## Results and Discussion

**Figure 1:**
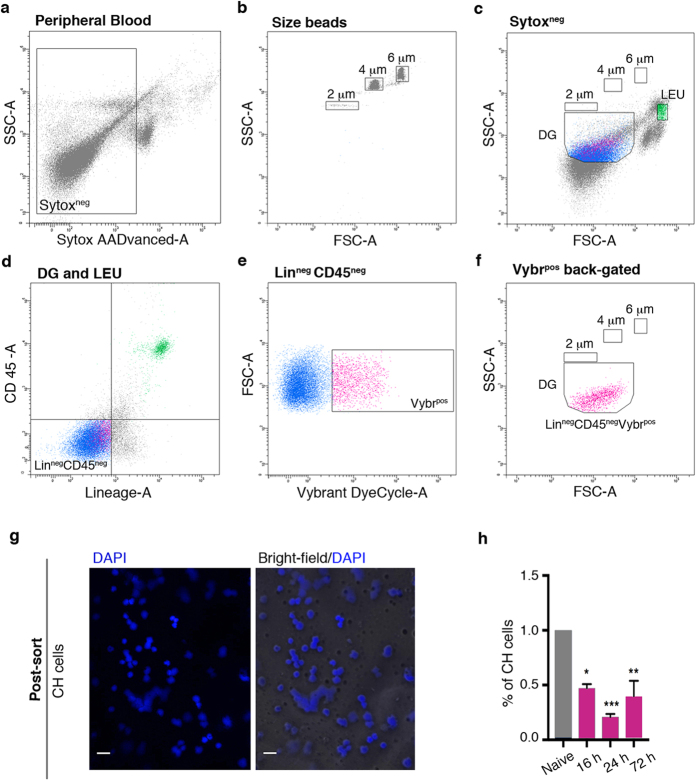
Small-sized Lin^neg^CD45^neg^Vybr^pos^ cells circulate in healthy mice and their percentage in the peripheral blood decreases after fracture induction. (**a**–**f**) Representative flow cytometry strategy used to identify Lin^neg^CD45^neg^Vybr^pos^Sytox^neg^ (CH) cells in the peripheral blood of C57Bl/6 mice. DG, dimensional gate; LEU, leukocytes (internal positive control). (**g**) DAPI staining (left panel) and overlay of DAPI and bright-field (right panel) on post-sorted CH cells. Magnification 60X; scale bar, 20 μm. (**h**) Histogram shows the percentage of CH cells identified in the peripheral blood of 20 naive mice (n = 5 independent experiments) and 60 fractured mice after 16, 24, and 72 hours post-damage induction (20 mice/considered time point; n = 5 independent experiments). Data are presented as mean ± SD. *P = 0.0105; **P = 0.013; ***P = 0.0004.

We attempted to isolate small-sized circulating progenitors from the peripheral blood of adult mice, using an innovative flowcytometry approach in order to exclude from the analysis subcellular particles, debris or nuclei expelled from erythroblasts during erythropoiesis^20^. The method entails the usage of beads with specific sizes to define appropriate dimensional gates in combination with two different DNA dyes (Sytox and Vybrant) to discriminate dead and viable cells. In particular, the Sytox dye specifically stains dead cells, as it easily penetrates cells with compromised plasma membranes and binds with a high affinity nucleic acids, whereas Vybrant permeates the viable cell membrane and emits fluorescence upon binding to double-stranded DNA. After doublet discrimination (Supplementary Fig. S1b) only Sytox-negative (Sytox^neg^) events were sorted and further analyzed (Fig. 1a). We defined cut-off gates of 2, 4 and 6 μm using microsphere suspensions of the appropriate dimensions (Fig. 1b), which ultimately allowed to delineate a specific dimensional gate (DG) within the Sytox^neg^ events (Fig. 1c). To successfully discriminate small-sized circulating progenitors, with a Lineage-negative, CD45-negative (Lin^neg^CD45^neg^) phenotype (Fig. 1d) from vesicles, cell-fragments and debris, we used the DNA dye Vybrant (Fig. 1e). The Lin^neg^CD45^neg^Vybr^pos^ (CH) cells fall exactly in the selected DG (Fig. 1f) as well as in the Sytox^neg^ gate (Supplementary Fig. S1a). Post-sorting characterization of Lin^neg^CD45^neg^Vybr^pos^ events indicated that CH cells were round-shaped and with a high nucleus/cytoplasm ratio, as indicated by the overlay of bright-field and DAPI staining (Fig. 1g). A mouse model of femur fracture was applied to test whether circulating CH cells could be recruited toward sites of injury to actively participate in the healing process. Fracture healing shares many similarities with soft-tissue healing with the exception of its ability to complete without the formation of a scar^21^. It represents a particularly appropriate model, since the injury microenvironmental cues could promote progenitor cell migration into a permissive microenvironment^22^. We determined the percentages of CH cells in the peripheral blood of the fractured animals at 16, 24, and 72 hours post-fracture and compared them to the percentage observed in non-injured (naive) mice. Several reports indicate that the number of different types of progenitor cells identified as circulating under extreme conditions increases in the peripheral blood following tissue injury^23^,^24^,^25^. Surprisingly, we observed that the percentage of CH cells was significantly lower in the peripheral blood of fractured mice as compared to naive mice, with maximum decrease after 24 hours and a trend back to physiologic conditions after 72 hours (Fig. 1h). These results indicate the existence of a specific population of progenitor cells constitutively circulating in the blood of healthy mice and suggest the possible occurrence of a homing event at the lesion site at an early stage of the healing process.

**Figure 2:**
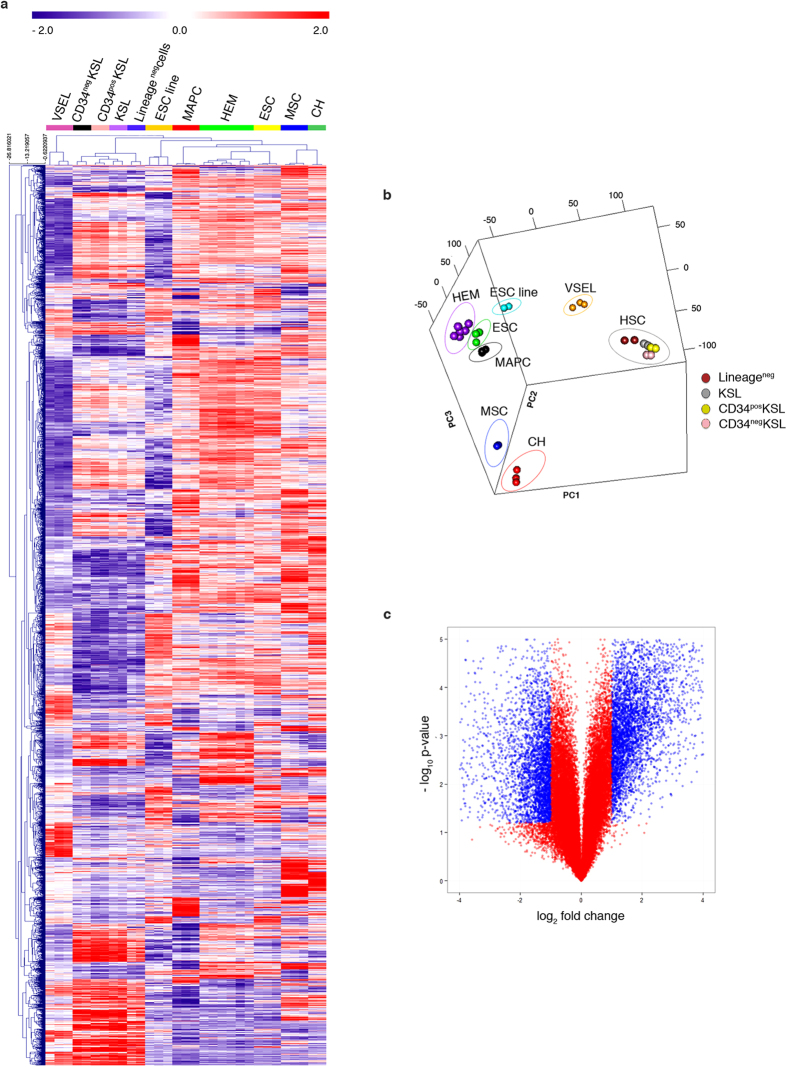
Characterization of CH cells by genome-wide microarrays. (**a**) Dendrograms show the hierarchical clustering, based on differentially expressed genes, of embryonic stem cell (ESC) line, ESC primary culture, c-kit^+^Sca-1^+^lineage marker^−^ cells (KSL), CD34^neg^ KSL, CD34^pos^ KSL, Lineage^neg^ cells, hemangioblasts (HEM), very small embryonic-like stem cells (VSEL), multipotent adult progenitor cells (MAPC), mesenchymal stem cells (MSC), and CH cells. (**b**) Principal component analysis of the 5000 genes with highest standard deviation for the samples mentioned in panel (**a**). (**c**) Volcano plot shows gene expression differences between CH cells and MSC. Log 2 gene ratios are plotted against negative log 10 P values.

**Figure 3:**
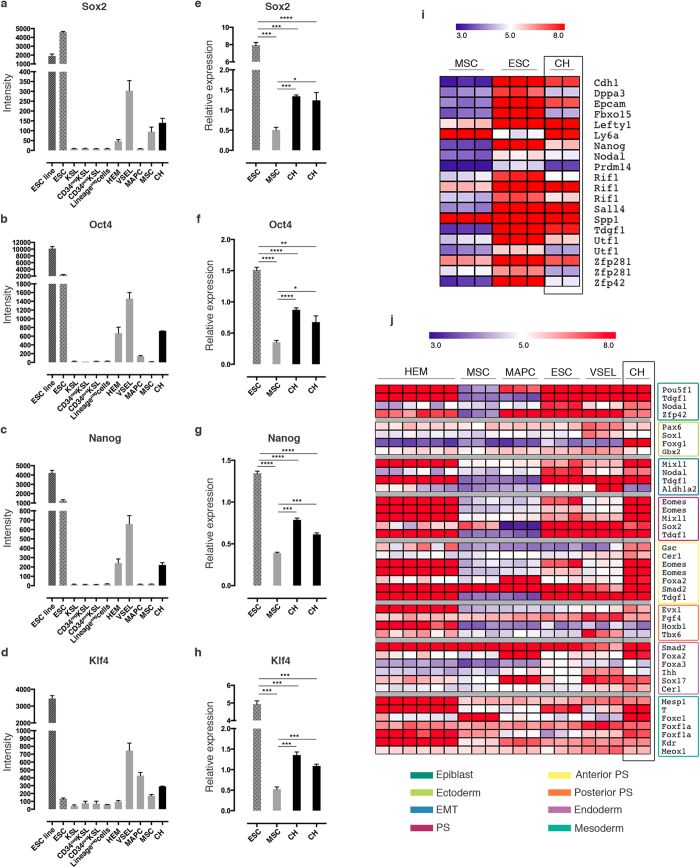
CH cells express key pluripotency genes and, characteristically, genes expressed at the epiblast stage during embryo development. (**a**–**d**) Analysis of the normalized array intensity levels of the stemness genes Sox2 (**a**), Oct4 (**b**), Nanog (**c**), and Klf4 (**d**) in the above mentioned cell populations. (e-h) The significant expression of the stemness genes in CH cells was confirmed by quantitative PCR analysis, comparing the gene expressions with MSC and ESC. (**e**) *P = 0.0142; ***P < 0.0007; ****P < 0.0001. (**f**) *P = 0.0225; **P = 0.0016; ****P < 0.0001. (**g**) ***P < 0.0003; ****P < 0.0001. (**h**) ***P < 0.0004. (**i**) Heatmap shows the differential expression of the main pluripotency-associated genes between ESC, MSC, and CH cells. (**j**) Heatmap shows the comparison of developmental-specific gene expressions among HEM, MSC, MAPC, ESC, VSEL, and CH. In the case of genes that were represented in the arrays by multiple probe sets, the signal intensity of all present probes is displayed. EMT, epithelial-mesenchymal transition; PS, primitive streak.

In order to assess CH cells’ identity in an unbiased manner, we performed global transcriptome analysis of FACS-purified populations from healthy mice. Gene expression profile of the purified cells was compared, after quantile normalization, to raw data of precursor cells characterized by varying stemness degree, which are available from the National Center for Biotechnology Information Gene Expression Omnibus (GEO). For the comparison, we specifically selected: (i) hematopoietic stem cells (HSCs) at different stages of differentiation, including hemangioblasts (HEM), primitive cells capable of both self-renewal and differentiation into mature blood cell lineages^26^,^27^; (ii) very small embryonic-like (VSEL) stem cells, pluripotent cells with a potential for tissue/organ regeneration^15^,^28^; (iii) embryonic stem cells (ESC), the “gold standard” of pluripotency^28^,^29^; (iv) multipotent adult progenitor cells (MAPC), a controversial cell population that has been reported to differentiate into tissues from different germ layers^30^; (v) mesenchymal stem cells (MSC), which can only differentiate into mesoderm lineages^31^. By hierarchical clustering analysis, we derived three clusters: the first included all the cells of the hematopoietic lineage (CD34^neg^ KSL, CD34^pos^ KSL, KSL, Lineage^neg^ cells); the second included all precursor cells (CH, ESC, HEM, MAPC, and MSC); and the last included just VSEL stem cells. CH cells formed a separate sub-cluster within the precursor cell cluster. MSC are the neighbors that cluster most closely to CH cells, even though the length of the dendrogram branch that separates the two populations indicates that they are clearly distinct (Fig. 2a). Principal component analysis (PCA) confirmed the unique expression profile of CH cells, that were separated from the other selected populations by projection in relation to the first 3 principal components representing a total variance of 69.5%, and specifically of 31.7% for PC1, 22.3% for PC2, and 15.5% for PC3 (Fig. 2b). Comparison between MSC and CH cells highlighted drastic differences in the expression profiles, as exemplified by the volcano plot (Fig. 2c). A rigorous statistical testing applying Significance Analysis of Microarray (SAM) indicated 902 up-regulated and 813 down-regulated genes (FDR 0%, 2-fold threshold) expressed in CH cells, when compared to MSC. Analysis of the expression pattern of main stemness genes in our datasets indicated that CH cells expressed high levels of *Sox2*, *Oct4*, *Nanog*, and *Klf4* (Fig. 3a–d). The latter result was further confirmed by quantitative PCR analysis (Fig. 3e–h). Sox2 and Oct4 synergistically activate Oct-Sox enhancers, which regulate the expression of pluripotent stem cell-specific genes^32^,^33^. Accordingly, heatmap analysis of main pluripotency-associated genes indicated that CH cells expressed *Utf1*, *Fbxo15*, *Lefty1*, *Nanog*, *Nodal*, *Dppa3*, *Prdm14 Spp1*, and *Zfp42*. The same genes were expressed by ESC, but not by MSC (Fig. 3i). Interestingly, CH cells expressed also high levels of *Cdh1*, *Epcam*, and *Sall4*, considered early ESC genes^34^, as well as *Tdgf1*, a critical gene for early embryonic development^35^. Analysis of the developmental signature using the LifeMap Discovery database^36^ indicated that CH cells expressed high levels of cell markers of the epiblast developmental stage, as well as of the downstream primitive streak (PS), anterior PS, and mesoderm stages (Fig. 3j).

**Figure 4:**
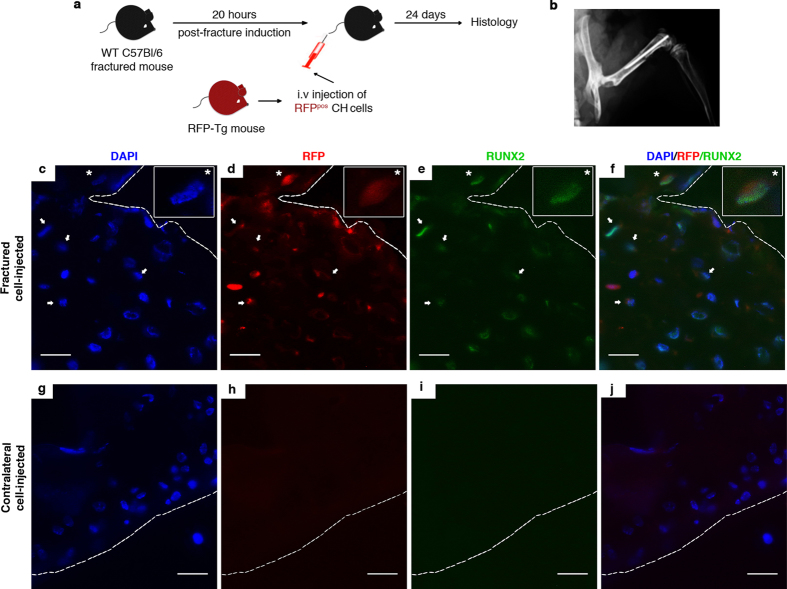
RFP^pos^ CH cell engraftment within the hard callus. (**a**) Schematic depicting systemic cell transplantation methodology. (**b**) Representative X-ray of the femoral osteotomy and internal fixation of the bone. **(c–f)** Triple fluorescence images showing signals from DAPI (blue) (**c**), endogenous RFP (red) (**d**), anti-Runx2 (green) (**e**) in the hard callus of fractured cell-injected mice. Overlap between DAPI, RFP, Runx2 is shown in panel **f**. White inset boxes in the panels show a higher magnification of a representative cell co-expressing DAPI, RFP, and Runx2 signals (white asterisk in the main panels). White arrows indicate RFP^pos^ Runx2^pos^ CH cells. **(g**–**j)** Triple fluorescence images showing signals from DAPI (blue) (**g**), endogenous RFP (red) (**h**), anti-Runx2 (green) (**i**) in the contralateral femur of fractured cell-injected mice. Overlap between DAPI, RFP, Runx2 is shown in panel (**j**). Magnification 40X, scale bar 20 μm.

Given the ability of CH cells to circulate in healthy conditions and their transcriptome profile, we tested their functional capacity to migrate toward wounded sites and to differentiate into a broad spectrum of tissue-specific cells. First, we isolated CH cells from the peripheral blood of transgenic naive mice that ubiquitously express the Red Fluorescent Protein (RFP) (Supplementary Fig. S2a–S2c), using the same flow cytometry strategy described in Fig. 1a–f. Vybr^pos^RFP^pos^ cells within the Lin^neg^CD45^neg^ gate were sorted (Supplementary Fig. S2d and S2e). Next, we intravenously (i.v.) injected hundred and fifty thousand RFP^pos^ CH cells in syngeneic WT mice 20 hours after femoral fracture (Fig. 4a,b). The injection time was chosen according to the decrease of CH cells observed in the blood stream of fractured mice (Fig. 1h). Twenty-four days after fracture induction, the minimal time required for the formation of a hard callus within the mouse fracture site^37^, the homing and engraftment of the transplanted cells (visualized by the RFP signal) were evaluated.

**Figure 5:**
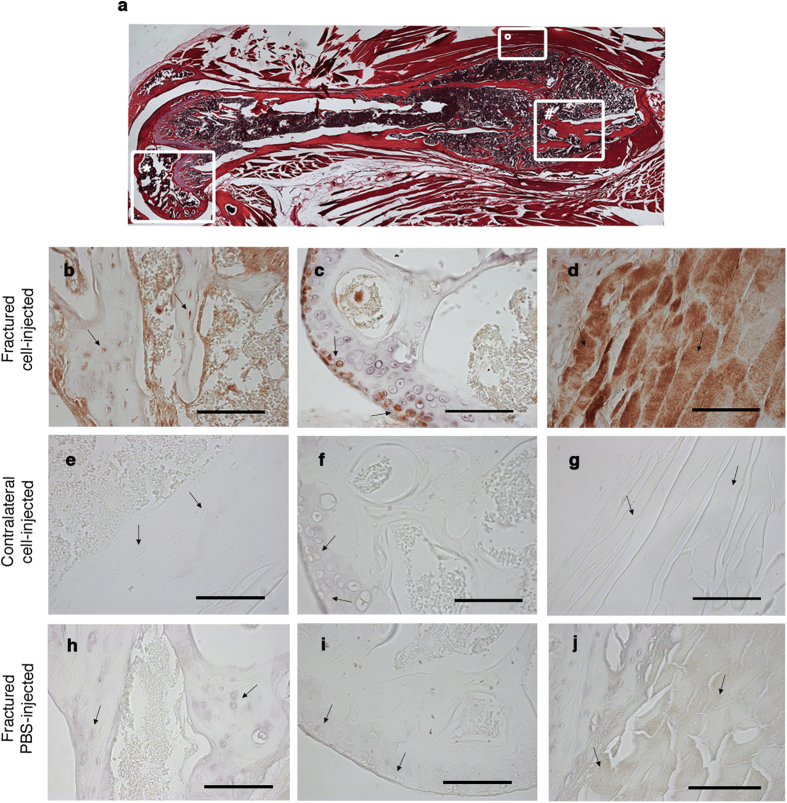
RFP^pos^ CH cells actively participate in tissue healing. (**a**) Serial hystology sections (magnification 5X) from the resulting fracture callus formed 24 days after the osteotomy. #hard callus; °muscle tissue surrounding the callus; *knee region. (**b**,**e**,**h**) Representative anti-RFP immunostaining of the hard callus region derived from fractured and cell-injected mouse (**b**), corresponding region of the contralateral paw of the same mouse (**e**), and the hard callus region derived from the fractured and PBS-injected mouse (**h**). Black arrows indicate osteoblasts within the bone matrix. **(c**,**f**,**i)** Representative anti-RFP immunostaining of the knee region derived from fractured and cell-injected mouse (**c**), contralateral paw of the same mouse (**f**), and fractured and PBS-injected mouse (**i**). Black arrows indicate articular chondrocytes. **(d**,**g**,**j)** Representative anti-RFP immunostaining of the muscle tissue derived from fractured and cell-injected mouse (**d**), contralateral paw of the same mouse (**g**), and fractured and PBS-injected mouse (**j**). Black arrows indicate muscle fibers. Magnification 40X, scale bar 100 μm.

The morphology examination of the healed fracture showed the general features of a hard callus, composed of multiple immature fragments of deposited bone intervened by fibrocartilaginous matrix (Fig. 5a). The immunofluorescence analysis of sections of the snap frozen tissue revealed the presence of several cells expressing an endogenous RFP signal and embedded in the bone matrix of the hard callus (Fig. 4c–f). Some of these RFP^pos^ cells co-expressed Runx2, a transcription factor known to be up-regulated during the early stage of osteogenesis^38^. Indeed, Runx2 is a key factor maximally expressed at initial stages of osteoblast maturation. RFP^pos^ Runx2^pos^ osteoprogenitors were interspersed with RFP^pos^ Runx2^neg^ cells, suggesting that some of the injected cells that were present in the bone matrix could have reached a more advanced osteogenic differentiation stage and lost the expression of that early marker (Fig. 4c–f). RFP^neg^ Runx2^pos^ cells were also detectable, possibly corresponding to recipient’s osteoprogenitors (Fig. 4c–f). No RFP signal was detected in the contralateral femurs of cell-injected mice (Fig. 4g–j). A more accurate visualization of the tissue was made by immunohistochemistry in decalcified paraffin embedded tissue sections looking for a marker of the late stage of osteogenesis such as osteocalcin. We confirmed the presence of fully differentiated tissue-specific RFP^pos^ cells in the matrix of the bone callus (Fig. 5b and Supplementary Fig. S3a–S3c). Other than the femur, additional tissues were damaged during the surgical procedure. These included the knee’s region as a consequence of the insertion of the needle stabilizer and the muscle tissue that was incised to access the femur (Fig. 5a). Thus, we evaluated the capacity of injected cells to migrate and engraft also in these additional damaged tissues. Tissue-specific RFP^pos^ cells were also identified in the articular cartilage (Fig. 5c and Supplementary Fig. S3d–S3f), as well as in the muscle tissue adjacent to the callus of the fractured paw (Fig. 5d, Supplementary Fig. S3g–S3i, and S4b). These data, along with the absence of RFP^pos^ cells in the circulation of mice 24 days post-fracture induction (Supplementary Fig. S2f–i), indicate the ability of CH cells of homing and engrafting in the damaged tissues and of giving rise to different types of tissue-specific cells. It is worthy of note that also by immunohistochemistry no RFP^pos^ cells were detected in the contralateral femurs of fractured and cell-injected mice (Fig. 5e–g). RFP^pos^ cells were not detected also in the fractured femurs of PBS-injected mice (Fig. 5h–j). CH cells positively contributed to the repair of the damaged tissues. Indeed, 42% of the articular chondrocytes and 45% of the osteoprogenitors expressed the RFP (Supplementary Fig. S4a).

Taken together, these results indicate that: (i) a rare and undifferentiated cell population involved in tissue healing is constitutively present in the peripheral blood of mice; (ii) the injury signals are sufficient to specifically direct their recruitment to the lesion site; (iii) the peculiar local microenvironment could determine their differentiation and appropriate integration into the specific tissue. Indeed, in our system the intrinsic characteristics coupled with the data from the whole genome analysis, lead to consider CH cells as a potential and novel source of connective tissue precursors able to differentiate toward different cell types, i.e. bone, cartilage, and muscle, in response to the tissue-specific microenvironments.

Our findings are in line with the idea that the specific recruitment of endogenous progenitors could enhance tissue repair or regeneration throughout the lifespan of the individual. Resident stem/progenitor cells function to maintain and, in some cases, repair tissues. Indeed, it has been described that when resident progenitors are depleted or dysfunctional, a premature tissue and aberrant repair occur^2^,^39^. This study raises the intriguing possibility that if we could understand how microenvironmental cues, as the ones generated by the injury event, enhance stem/progenitor cell function, we could then exogeneously manipulate these same mechanisms to promote tissue repair. Otherwise, this has been a very successful approach in the hematopoietic compartment, in which Granulocyte-Colony Stimulating Factor (G-CSF) is used clinically to mobilize hematopoietic precursors^40^.

The deep comprehension of the reciprocal interactions between microenvironmental signals and CH cells could lead to manipulate these mechanisms to promote tissue repair. If a comparable cell population were to be identified also in the human peripheral blood, we anticipate that it could be used in the future as an effector of therapeutic tissue regeneration as well as in the repair of tissue defects caused by disease or trauma.

## Methods

### Mice

C57Bl/6 (MHC H2b haplotype) mice were purchased from Charles River Laboratories (Calco, Italy). Red fluorescent protein-transgenic (RFP-Tg) mice (B6.Cg-Tg(CAG-DsRed*MST)1Nagy/J) were purchased from The Jackson Laboratory (Bar Harbor, MA, USA). We used female and male mice between 5 and 8 weeks of age. Mice were bred and maintained at the Animal Facility of “IRCCS Azienda Ospedaliera Universitaria San Martino – IST Istituto Nazionale per la Ricerca sul Cancro”. All animal procedures were approved by the“IRCCS Azienda Ospedaliera Universitaria San Martino – IST Istituto Nazionale per la Ricerca sul Cancro” Ethical Committee and performed in accordance with the national current regulations regarding the protection of animals used for scientific purpose (D. Lgs. 4 marzo 2014, n. 26, legislative transposition of Directive 2010/63/EU of the European Parliament and of the Council of 22 September 2010 on the protection of animals used for scientific purposes).

### Isolation of Circulating Healing (CH) cells

CH cells were isolated from the peripheral blood (PB) of non-fractured (naïve) WT and RFP-Tg mice, and from fractured WT mice at different times post-fracture induction (16, 24, 72 hours). Mice were anesthetized with an intraperitoneal injection of 100 μl/20 gr b.w. of stock solution containing ketamine HCl (100 mg/kg), xylazine (10 mg/kg), and PB was harvested from the retro-orbital vein and collected into heparin-coated tubes. Whole PB samples were lysed twice using a BD Pharm Lyse (BD Biosciences, Milan, Italy). The peripheral blood mononuclear cells (PBMCs) derived from each experimental group were pooled together (five mice/group) and stained for Fluorescence-activated cell sorting (FACS) analysis. Experiments were repeated at least four times.

### FACS and cell sorting

To determine the amount of Lin^neg^CD45^neg^Vybr^pos^ (CH) cells, PBMCs derived from fractured and naive mice were collected in FACS buffer (PBS containing 2% heat-inactivated foetal bovine serum) and stained for 10 min at 4 °C with Lineage Cell Detection Cocktail-Biotin antibody (Lin), (Miltenyi Biotec, Bergish Gladbach, Germany). A Streptavidin PE-Cy7 (eBioscience, San Diego, CA, USA) was used for indirect staining to detect the biotinylated antibody cocktail. After washing, cells were stained with APC rat anti-mouse CD45 antibody (clone 30-F11) (BD Biosciences). A double staining with Vybrant DyeCycle Green Stain and Sytox AADvanced Dead Cell Stain (Molecular Probes, Milan, Italy) was used to discriminate debris, live and dead cells. A set of microsphere suspensions (2, 4, 6 μm) (Molecular Probes) was used as size references.

CH cells were sorted, with high purity mask, from naive and RFP-Tg mice. All experiments were performed on BD FACSAria II. Data were analyzed using BD FACSDiva software.

### Mouse Femoral Fracture model and CH cell injection

All surgical procedures were performed under anesthesia and normal sterile conditions. Briefly, mice were anesthetized using the same regimen above described and a lateral parapatellar knee incision on the right hindlimb was made in WT C57Bl/6 mice to expose the distal femoral condyle. A medullary rod was placed via the intercondylar fossa and the stabilizer needle (27-gauge) was inserted. A transverse mid-diaphyseal femoral shaft fracture was created, and the skin incision closed with 5–0 nylon sutures. The mice received post-operative analgesia (Buprenorphine 0.1 mg/kg SC q 12 h), and unprotected weight bearing was allowed immediately post-operation. Postoperative radiographic assessment was performed and the mice with incomplete fracture were excluded from further analysis. Hundred and fifty thousand RFP^pos^ CH cells were intravenously (i.v.) injected in syngeneic WT mice 20 hours post-fracture induction.

### Histology and Immunofluorescence analysis

For isolated single cell analysis, CH cells were sorted onto glass slides at 30.000 events per slide, air-dried and fixed with 1:1 Acetone/Methanol fixative (−20 °C, 10 minutes). The DNA of sorted cells was counter-stained with DAPI (Abbott Molecular Inc, Des Plaines, IL, USA) to trace the outline of the nuclei. Image of cells were captured using an Axiovert 200 M microscope (Zeiss, Germany).

Bilateral femurs derived from fractured and cell-injected mice were harvested and embedded in OCT compound, snap frozen in liquid nitrogen, and stored at −80 °C for immunofluorescence analysis. Mouse femurs in OCT blocks were sectioned, and 5 μm serial sections were collected on slides followed by fixation with 4% formaldehyde at room temperature for 15 minutes. After washing with PBS, sections were covered with ice-cold 100% methanol at −20 °C for 5 minutes and treated with a blocking buffer (1× PBS/5% normal goat serum/0.3% Triton X-100) for 60 minutes. Immunofluorescence staining was performed using a Runx2 rabbit mAb (Cell Signaling Technology, Milano, Italy) followed by a Alexa Fluor 633-conjugated goat anti-rabbit IgG Alexa Fluor 633-conjugated secondary antibody (Thermo Scientific, Rockford, IL, USA). A DAPI solution was applied for 5 minutes for nuclear staining. Images were captured using an Axiovert 200 M microscope (Zeiss, Germany).

For tissue immunohistochemistry analysis, paws of fractured and control mice were decalcified with a decalcifying solution (code 52544, prepared at “Farmacia Azienda Ospedaliera San Martino”, Genova, Italy) and embedded in paraffin using standard histological techniques. Four-micrometer serial sections were cut longitudinally. Sections were stained with H/E. To detect the presence of RFP-positive CH cells within fractured or contralateral femurs, knee, and muscle, 4-μm sections were treated with rabbit polyclonal anti-RFP antibody (1:100) (Molecular Probes Europe BV, Leiden, The Netherlands), followed by EnVision+ System-HRP Labelled Polymer anti-rabbit antibody (Dako, Carpinteria, CA, USA). To detect the presence of cartilaginous and bone tissues, we used a polyclonal rabbit anti-mouse collagen type II antibody (1:300) (Chemicon International, Temecula, CA), and a polyclonal rabbit anti-bovine bone Osteocalcin (1:200) (provide by Larry Fisher, NIH, Bethesda, USA) respectively. Muscle fibers close to the hard callus were detected using a rabbit polyclonal anti-dystrophin antibody (Santa Cruz Biotechnology, Heidelberg, Germany). RFP+/Dystrophin+ muscle fibers were also detected by double immunohistochemistry revealing the HRP-conjugated secondary antibody used for the anti-RFP antibody with the 3,3′ diamino benzidine (DAB) (Dako, Carpinteria, CA, USA) in brown and the AP-conjugated secondary antibody used for the anti-dystrophin antibody with BM purple (Sigma Aldrich, Milano, Italy) in light blue. Negative controls with pre-immune serum were run in parallel. Images were captured by transmitted light microscopy with an Olympus C3030 digital camera and Camedia Master Olympus software.

### Cell quantification analysis

Six random fields per callus and per knee of fractured and cell-injected mice were analyzed by using ImageJ Tool to calculate the number of RFP^pos^ cells within the outlined bone matrix area and within the outlined articular cartilage, respectively. The outlined areas were referred as region of interest (ROI). Results were expressed as percentage of RFP^pos^ and RFP^neg^ cells in a ROI of 0.06 mm^2^ for the hard callus and in a ROI of 0.02 mm^2^ for the knee.

### RNA extraction and Real-Time PCR analysis

Total RNA was extracted from CH cells using the RNeasy® Micro Kit (Qiagen, Milano, Italy) according to the manufacturer’s instructions. For the reverse transcription (RT) reactions and cDNA synthesis, 2 μg of total RNA was used in the Omniscript® RT kit (Qiagen, Milano, Italy) following the manufacturer’s instructions. The gene expression analysis was performed by Real Time-PCR using the PE ABI PRISM 7700 sequence detection system (Perkin-Elmer, Waltham, MA) and SYBR Green (Applied Biosystems, Foster City, CA). The sequences of forward and reverse primers were as follows: Oct4, 5′-ACATCGCCAATCAGCTTGG-3′ and 5′AGAACCATACTCGAACCACATCC-3′; Nanog, 5′-AAGATGCGGACTGTGTTCTC-3′ and 5′-CGCTTGCACTTCATCCTTTG-3′; Sox2, 5′-AAGGGTTCTTGCTGGGTTTT-3′ and 5′-AGACCACGAAAACGGTCTTG-3′; Klf4, 5′-GAAGACCAGGATTCCCTTGA-3′ and 5′- CCAAGCACCATCATTTAGGC -3′. The expression of GAPDH (5′-TGTGTCCGTCGTGGATCTG-3′ and 5′-GATGCCTGCTTCACCACCTT-3′) was examined as endogenous control. Relative transcript levels were calculated from the relative standard curve constructed from stock cDNA dilutions and divided by the target quantity of the calibrator following manufacturer’s instructions.

### Gene expression profiling

Gene expression profiles were generated using Affymetrix MG 430.2 microarrays. Total RNA was extracted from approximately 100.000 CH cells. cRNA for hybridisation was prepared using the GeneChip® 3′ IVT PLUS Reagent Kit and following the instructions provided by Affymetrix (Santa Clara, CA). Hybridisation and scanning were performed following standard procedures recommended by Affymetrix. The data were deposited in the Gene Expression Omnibus repository (www.ncbi.nlm.nih.gov/geo/), accession number: GSE64835. For data analysis CH cell array data were normalised together with raw data from GSE2981, GSE27787, GSE43042, GSE6933, GSE47935 datasets using RMA algorithms with quantile normalisation implemented in R/BioConductor. Quantile normalization forces the data of all chips analysed into the same distribution thereby minimising batch effects and is therefore best suited to compare data from different sources. Significantly differentially expressed genes were identified after filtering of non-expressed genes using Significance Analysis of Microarrays implemented in TMEV and visualized by hierarchical clustering after removal of genes with expression values below 5 (log2) throughout the entire dataset using Pearson correlation distance and average linkage. Principal component analysis was performed in R (library rgl) on the 5000 genes with highest standard deviation. Volcano plots were generated in R (library a4Base). Absolute expression values were used for generating heatmaps for germ layer specific and pluripotency associated genes.

### Statistical analysis

One-way ANOVA and Tukey’s multiple comparisons test were applied to calculate the statistical significance of percentage of CH cells isolated from the different experimental groups by FACS. An unpaired two-tailed Student’s t-test was used to evaluate the statistical significance of Sox2, Oct4, Nanog and Klf4 expression date obtained using real time-PCR after calculation of the averaged gene/internal GAPDH ratio. Statistical significance was set at p < 0.05. All statistical analyses were performed using GraphPad Prism Version 6.0a (GraphPad Software, La Jolla, CA, USA).

## Additional Information

**How to cite this article**: Sicco, C. L. *et al.* Identification of a New Cell Population Constitutively Circulating in Healthy Conditions and Endowed with a Homing Ability Toward Injured Sites. *Sci. Rep.* **5**, 16574; doi: 10.1038/srep16574 (2015).

## Supplementary Material

Supplementary Information
