# Supplementary material for: Identification of a New Cell Population Constitutively Circulating in Healthy Conditions and Endowed with a Homing Ability Toward Injured Sites
Source: Sci Rep. 2015 Nov 12;5:16574. doi: 10.1038/srep16574 (PMC4642305; doi:10.1038/srep16574)
Supplement: Supplementary Information [file srep16574-s1.pdf]

Supplementary Information

Identification of a New Cell Population Constitutively Circulating in Healthy Conditions and Endowed with a Homing Ability Toward Injured Sites.

Claudia Lo Sicco[1,2,3], Roberta Tasso[1,2,3 *], Daniele Reverberi[2], Michele Cilli[2], Ulrich Pfeffer[2] & Ranieri Cancedda[1,2]

[1]Department of Experimental Medicine (DIMES), University of Genova, Genova, 16132, Italy

[2]A.O.U. San Martino-IST, National Cancer Research Institute, Genova, 16132, Italy

[3] These authors contributed equally to this study.

[*] Correspondence: robertatasso@gmail.com

[*] Work telephone number: +39-010-5558319

Supplementary Figure 1

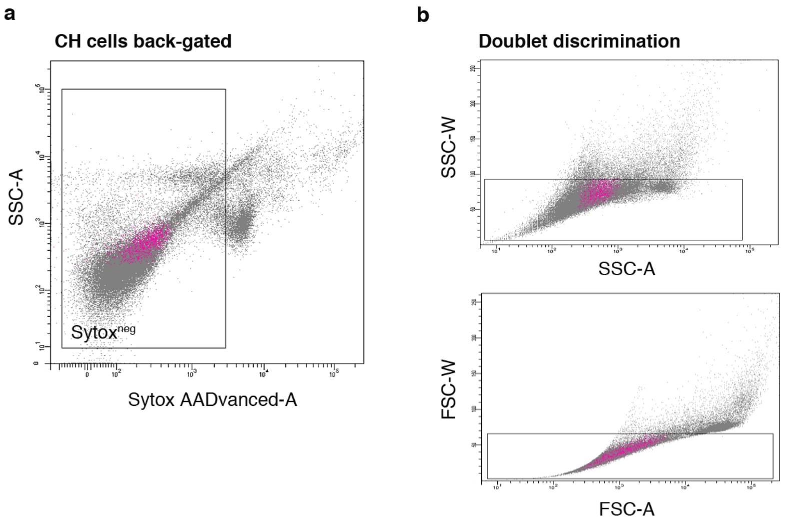

Supplementary Fig. S1. Back-gating of Lin^{neg}CD45^{neg}Vybr^{pos} (CH) cells. (**a**) Representative flow cytometric analysis of CH cells back-gated in the dot plot of side scatter (SSC-A) versus Sytox AADvanced dye. (**b**) Doublet discrimination. CH cells are shown in fuchsia.

Supplementary Figure 2

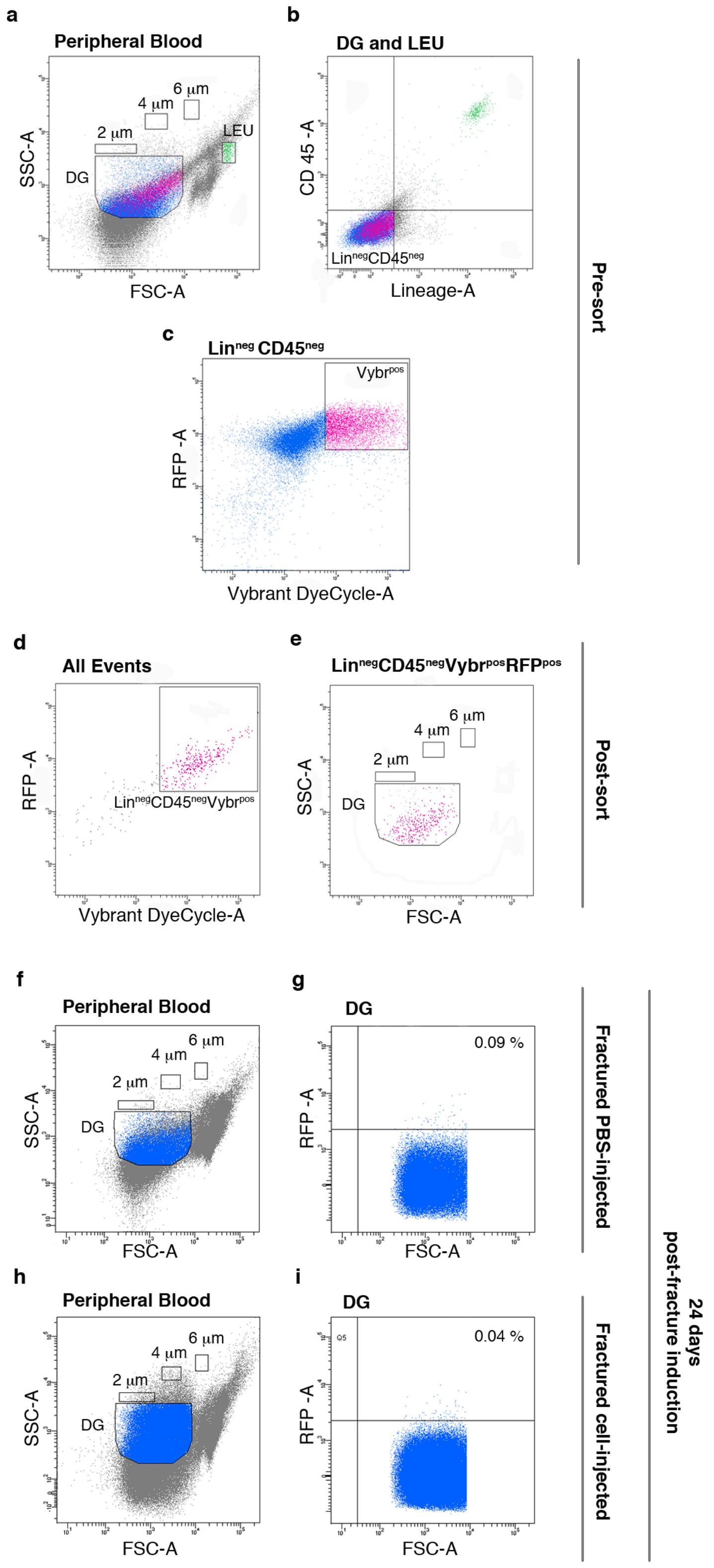

Supplementary Fig. S2. Identification of RFP^{pos} CH cells. (**a-c**) Representative flow cytometry

strategy used to identify $Lin^{neg}CD45^{neg}Vybr^{pos}$ (CH) cells in the peripheral blood of C57Bl/6 transgenic for the ubiquitous RFP expression ($Lin^{neg}CD45^{neg}Vybr^{pos}RFP^{pos}$). DG, dimensional gate; LEU, leukocytes. (**d, e**) Re-analysis of sorted RFP^{pos} CH cells based on intrinsic fluorescence (Vybrant and RFP)and physical parameters. (**f-i**) Representative flow cytometry analysis of RFP^{pos} cells present in the peripheral blood of fractured PBS-injected mice (**f,g**) and fractured cell-injected mice (**h,i**) 24 days post-fracture induction.

Supplementary Figure 3

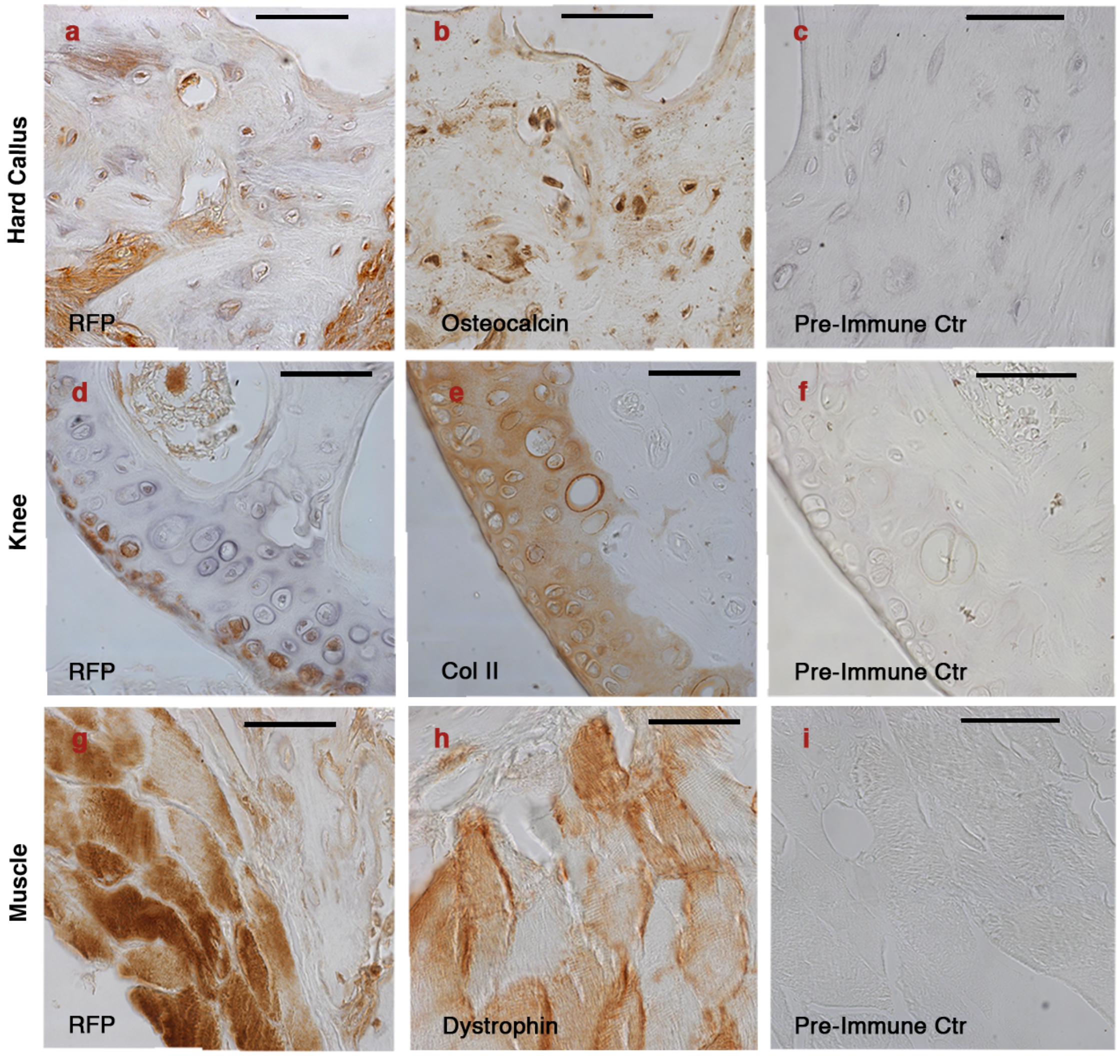

Supplementary Fig. S3. RFP^{pos} CH cells differentiate into tissue-specific cells. (**a-c**) Serial sections of hard callus formed after 24 days in fractured and cell injected mice. (**a**) Representative immunohistochemical analysis conducted using a specific anti-RFP antibody; (**b**) Representative immunohistochemical analysis conducted using a specific anti-Osteocalcin antibody; (**c**) Negative control with pre-immune serum. (**d-f**) Serial sections of the knee region of fractured and cell injected mice. (**d**) Representative immunohistochemical analysis conducted using a specific anti-RFP antibody; (**e**) Representative immunohistochemical analysis conducted using a specific anti-

type II Collagen antibody; (**f**) Negative control with pre-immune serum. (**g-i**) Serial sections of muscle tissue surrounding the callus formed after 24 days in fractured and cell injected mice. (**g**) Representative immunohistochemical analysis conducted using a specific anti-RFP antibody; (**h**) Representative immunohistochemical analysis conducted using a specific anti-Dystrophin antibody; (**i**) Negative control with pre-immune serum. Each panel is the merged result of 6 serial microscope images. Magnification 100X; scale bar, 50 μm.

Supplementary Figure 4

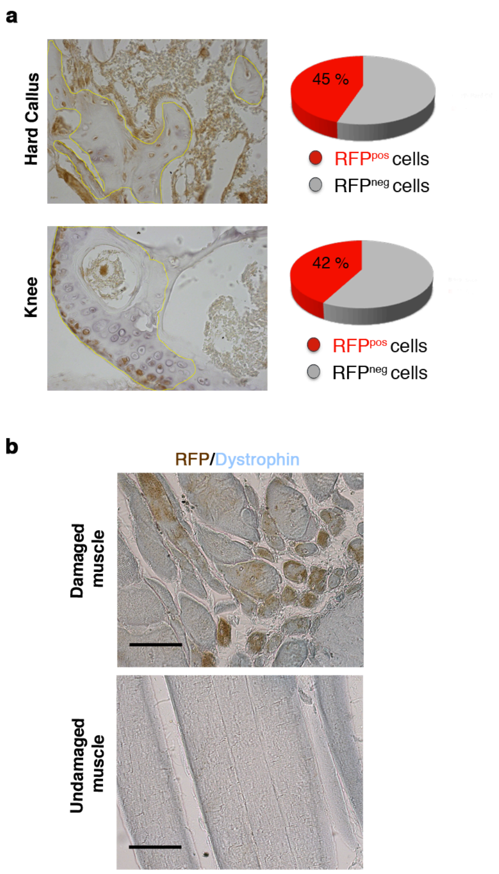

Supplementary Fig. S4. Presence of CH cells in the damaged tissues. (**a**) Quantification of RFP^{pos} and RFP^{neg} cells engrafted in the hard callus (upper panels) and in the knee region (bottom panels). The yellow lines indicate two representative regions of interest (ROI). (**b**) Representative double immunohistochemical analysis conducted using a specific anti-dystrophin antibody revealed in light blue and a specific anti-RFP antibody revealed in brown. Muscle tissue adjacent to the hard callus (upper panel) and out of the hard callus region (bottom panel) were analyzed. Magnification 40X; scale bar, 50 mm.
